# Estimation of the water quality of a large urbanized river as defined by the European WFD: what is the optimal sampling frequency?

**DOI:** 10.1007/s11356-016-7109-z

**Published:** 2016-07-25

**Authors:** Lauriane Vilmin, Nicolas Flipo, Nicolas Escoffier, Alexis Groleau

**Affiliations:** 1 Geosciences Department, MINES ParisTech, PSL Research University, 35 rue Saint Honoré, 77305 Fontainebleau, France; 2 Present Address: Department of Earth Sciences — Geochemistry, Faculty of Geosciences, Utrecht University, P.O. Box 80021, 3508TA Utrecht, The Netherlands; 3 Institut de Physique du Globe de Paris, Sorbonne Paris Cité, Univ Paris Diderot, UMR 7154 CNRS, 75005 Paris, France; 4 Present Address: Stream Biofilm and Ecosystem Research Laboratory, École Polytechnique Fédérale de Lausanne (EPFL), Station 2, CH-1015 Lausanne, Switzerland

**Keywords:** European water framework directive, Optimal sampling frequency, River water quality assessment, Orthophosphate, Inorganic nitrogen, Chlorophyll *a*, Oxygen, Hydro-biogeochemical modeling

## Abstract

Assessment of the quality of freshwater bodies is essential to determine the impact of human activities on water resources. The water quality status is estimated by comparing indicators with standard thresholds. Indicators are usually statistical criteria that are calculated on discrete measurements of water quality variables. If the time step of the measured time series is not sufficient to fully capture the variable’s variability, the deduced indicator may not reflect the system’s functioning. The goal of the present work is to assess, through a hydro-biogeochemical modeling approach, the optimal sampling frequency for an accurate estimation of 6 water quality indicators defined by the European Water Framework Directive (WFD) in a large human-impacted river, which receives large urban effluents (the Seine River across the Paris urban area). The optimal frequency depends on the sampling location and on the monitored variable. For fast varying compounds that originate from urban effluents, such as PO$_{4}^{3-}$, NH$_{4}^{+}$ and NO$_{2}^{-}$, a sampling time step of one week or less is necessary. To be able to reflect the highly transient character of bloom events, chl *a* concentrations also require a short monitoring time step. On the contrary, for variables that exert high seasonal variability, as NO$_{3}^{-}$ and O _2_, monthly sampling can be sufficient for an accurate estimation of WFD indicators in locations far enough from major effluents. Integrative water quality variables, such as O _2_, can be highly sensitive to hydrological conditions. It would therefore be relevant to assess the quality of water bodies at a seasonal scale rather than at annual or pluri-annual scales. This study points out the possibility to develop smarter monitoring systems by coupling both time adaptative automated monitoring networks and modeling tools used as spatio-temporal interpolators.

## Introduction

Freshwater represents a very small fraction of the Earth’s total water resources. Preserving its quality, while meeting the needs of human activities (drinking water production, industry, irrigation, *etc.*), has been one of the main challenges of the last decades. Water quality is a broad term, though, that can notably comprise the biological, physical or chemical statuses of a water body. It is controlled by various environmental processes that can be affected by human activities. It is therefore an important task to evaluate the quality of water bodies, in order to assess the impacts of human activities and the effects of remediation strategies.

The estimation of the quality status of water bodies is based on the comparison of various indicators with fixed thresholds. These indicators correspond to statistical criteria calculated on measured time series of water quality variables. For a given water body, the measured data may be available at one or several locations, and at various frequencies. It generally originates from the analysis of grab/discrete samples, which are carried out at rather low frequency (monthly).

In Europe, water quality standards are fixed by the European Water Framework Directive (WFD, Parliament Council of the European Union (2000)). The quality status is assessed through both ecological and chemical statuses. We focus here on variables of the ecological status. In the scope of the WFD, the indicators used to evaluate the quality of a water body in terms of PO$_{4}^{3-}$, NH$_{4}^{+}$, NO$_{2}^{-}$, NO$_{3}^{-}$ and chlorophyll *a* is the annual 90 % concentration quantile, while the 10 % quantile is used for dissolved O _2_. These indicators were chosen to traduce the environment’s sensitivity to concentration peaks (or O _2_ drops) (Polus et al. 2010). The water quality standards define 5 quality classes (Table 1). It is worth noting that the thresholds defining these classes may vary according to the type of water body (i.e., natural water bodies versus water bodies that are strongly modified by human activities). The quality status of a water body is then defined according to the result of the most downgrading indicator. The initial objective of the WFD was to reach the good status of water bodies by 2015. However, this deadline has been shifted to 2021 or 2027 for strongly modified water bodies (i.e., subject to strong anthropic pressures), for which good quality was not achieved (Direction régionale de l’Environnement Ile-de-France 2010).

**Table 1:** Ranges of the different water quality statuses for the studied indicators (Ministère de l’Écologie, du Dèveloppement durable et de l’Énergie 2012)

Indicator	Unit	Quality status				
		Very good	Good	Medium	Poor	Bad
$[\text {PO}_{4}^{3-}$] _90_	mgPO$_{4}^{3-}\cdot \textit {L}^{-1}$	0–0.1	0.1–0.5	0.5–1	1–2	>2
$[\text {NH}_{4}^{+}$] _90_	mgNH$_{4}^{+}\cdot \textit {L}^{-1}$	0–0.1	0.1–0.5	0.5–2	2–5	>5
$[\text {NO}_{2}^{-}$] _90_	mgNO$_{2}^{-}\cdot \textit {L}^{-1}$	0–0.1	0.1–0.3	0.3–0.5	0.5–1	>1
$[\text {NO}_{3}^{-}$] _90_	mgNO$_{3}^{-}\cdot \textit {L}^{-1}$	0–10	10–50	–	–	–
[chl *a*]_90_	*μ*gchl *a* ⋅*L* ^−1^	0–10	10–60	60–120	120–240	>240
[O_2_] _10_	mgO$_{2}\cdot \textit {L}^{-1}$	>8	6–8	4–6	3–4	0–3

One of the major challenges in the estimation of the quality of a water body is how to represent this water body by a limited number of sampling points (Carstensen 2007), and thus achieve a reliable assessment of its quality status. This raises the question of which spatial and temporal resolutions are necessary for a good assessment of the water quality. The WFD provides no precise guidance to address this question. However, it recommends to calculate the quality indicators for a minimum period of 3 years and with a minimum of six measurements per year for each water body. The French instructions followed these recommendations (Ministère de l’Écologie du Développement durable et de l’Énergie 2013). Location of sampling is a critical step in the design of monitoring networks (Dixon et al. 1999; Do et al. 2012). Polus et al. (2010) have shown the importance of spatial resolution for the estimation of different water quality criteria. The data from two stations of a same water body (as defined in the scope of the WFD) can lead to the estimation of quality indicators, which correspond to different water quality statuses. These spatial heterogeneities can notably be due to anthropogenic effluents, which are not taken into account for the spatial definition of water bodies (Wasson et al. 2003).

Besides the spatial resolution of the monitoring data, the temporal resolution is also important. The measurement time step can indeed impact the estimation of the various water quality indicators. The need for high frequency monitoring dedicated to the understanding of hydrological and biogeochemical processes—that often occur in minute/hour rather than weekly/monthly time scales (Tomlinson and De Carlo 2003)—is well recognized (Kirchner et al. 2004; Harris and Heathwaite 2005; Kirchner 2006; Hart and Martinez 2006; Horsburgh et al. 2010). A 60-day measurement time step is most often insufficient to fully capture the variability of one water quality variable. Several authors have focused on the impact of the sampling frequency on the estimation of fluxes (Ferrant et al. 2012; Wade et al. 2012; Moatar et al. 2013). For instance, Ferrant et al. (2012) showed that, for frequencies over one day, NO$_{3}^{-}$ fluxes were overestimated during flood periods. Moatar et al. (2013) highlighted the fact that suspended sediment fluxes calculated on time series at a monthly sampling frequency could display ±100 % uncertainties. The estimation of statistical criteria and of the quality status of a water body from low frequency measurements may also be flawed (Bernard-Michel and de Fouquet 2005; Bernard-Michel 2006). This may particularly be true in the case of strongly anthropized systems that are characterized by frequent transient events, as summarized in the context of the “urban stream syndrome” (Walsh et al. 2005).

Yet, given the high cost of environmental monitoring, budgetary resources need to be considered in the design of monitoring strategies (Lettenmaier 1979; Strobl and Robillard 2008). The optimization of measurement strategies is therefore essential, in order to maximize their cost-effectiveness (Dixon and Chiswell 1996). The sampling frequency is very important in the design of monitoring strategies. Indeed, it affects not only the precision of the information that is extracted from the collected data, but also operational costs (Khalil and Ouarda 2009). Naddeo et al. (2013) showed that it constitutes a promising parameter to be optimized. As summarized by Sanders and Adrian (1978), at first, sampling frequencies were mostly determined based on the ability to detect violations of water quality standards or extreme events as pollution spills. Requirements of surveillance networks were later oriented toward the assessment of ambient water quality conditions. In this goal, Sanders and Adrian (1978) proposed a method to reach a confidence interval width for the mean of the random component of a measured time series. Various studies on surface or groundwater monitoring were carried out using similar statistical methods (Lo et al. 1996; Zhou 1996). More recently, the entropy theory method, which quantifies the amount of transinformation within a dataset (Yang and Burn 1994), was applied for the assessment and the design of monitoring strategies (Karamouz et al. 2009; Mahjouri and Kerachian 2011).

In the present work, we propose a new methodology, based on the multiple re-sampling of high-resolution simulation results from a physically-based biogeochemical model, to assess optimal sampling frequencies for the monitoring of six variables: orthophosphate (PO$_{4}^{3-}$), ammonium (NH$_{4}^{+}$), nitrite (NO$_{2}^{-}$), nitrate (NO$_{3}^{-}$), chlorophyll *a* (chl *a*), and dissolved oxygen (O _2_) concentrations. The methodology is applied to a large human-impacted river system: the Seine River from the Paris urban area to the entrance of its estuary. The hydro-biogeochemical functioning of this system is simulated along this 220 km river stretch at a 15-min time step over a 6-year period (2007–2012). A comparison of model outputs with available high frequency data is performed in order to ensure that the model is reliable at small time scales and can be used as a “high frequency estimator” of the water quality of the Seine River regarding the studied variables. The analysis of the modeled variables’ time series allows for the quantification of the effect of the sampling strategy on the estimation of the corresponding WFD quality indicators, both in terms of sensitivity to spatial heterogeneities and to the temporal resolution of the sampling. The hydro-biogeochemical modeling tool is used to assess optimal sampling time steps for an accurate estimation of the quality status of the river along the studied stretch as defined by the WFD.

## Simulation of the water quality of the Seine River from the Paris urban area to the entrance of the estuary

### Assessment of concentrations and variability of water quality variables

#### The hydro-biogeochemical ProSe model

The different studied variables (PO$_{4}^{3-}$, NH$_{4}^{+}$, NO$_{2}^{-}$, NO$_{3}^{-}$, chl *a*, O _2_) are assessed at fine spatial and temporal scales (500 m cells, 15 min time step) with the ProSe model (Even et al. 1998; 2004; 2007; Flipo et al. 2004; 2007). The ProSe model simulates the hydro-biogeochemical functioning of river networks and their response to anthropogenic pressure in transient state. It is composed of three modules:

- a hydrodynamic module, which solves the 1D shallow water equations,
- a transport module, which simulates advection and dispersion of particulate and dissolved compounds,
- a biogeochemical module.

The biogeochemical module is based on the rive conceptual model (Billen et al. 1994; Garnier et al. 1995). It simulates the processes affecting the cycles of carbon, major nutrients, and dissolved oxygen in both benthic and water-column compartments of the river system. The dissolved and particulate exchanges between these compartments are also simulated (Flipo et al. 2004). The simulation of hydro-sedimentary processes, phosphorus sorption, and nitrogen dynamics has been recently updated to take into account the effect of recent improvements in waste water treatment plant (WWTP) technologies (Raimonet et al. 2015; Vilmin et al. 2015b, a).

#### Estimation of the annual variability of water quality variables

The contribution of the seasonal variability to the total variability is assessed for the different studied variables in order to check if any link exists with the estimated optimal sampling frequencies. This is done through a variographic analysis. As described in Vilmin et al. (2015b), a multi-component variographic model (Chilès and Delfiner 1999; de Fouquet et al. 2007; Polus et al. 2011) is fitted to each variable’s temporal variogram at different stations. This allows for the description of the variable as a linear combination of different temporal structures, including a periodic component that represents seasonal variations. By fitting variographic models to the variograms, the proportions of the total variability due to each of the temporal structures can be quantified. The proportion of the annual variability in the total variabilities of the studied variables is thereby assessed at several locations (Table 3).

### Application to the Seine River

**Fig. 1:**
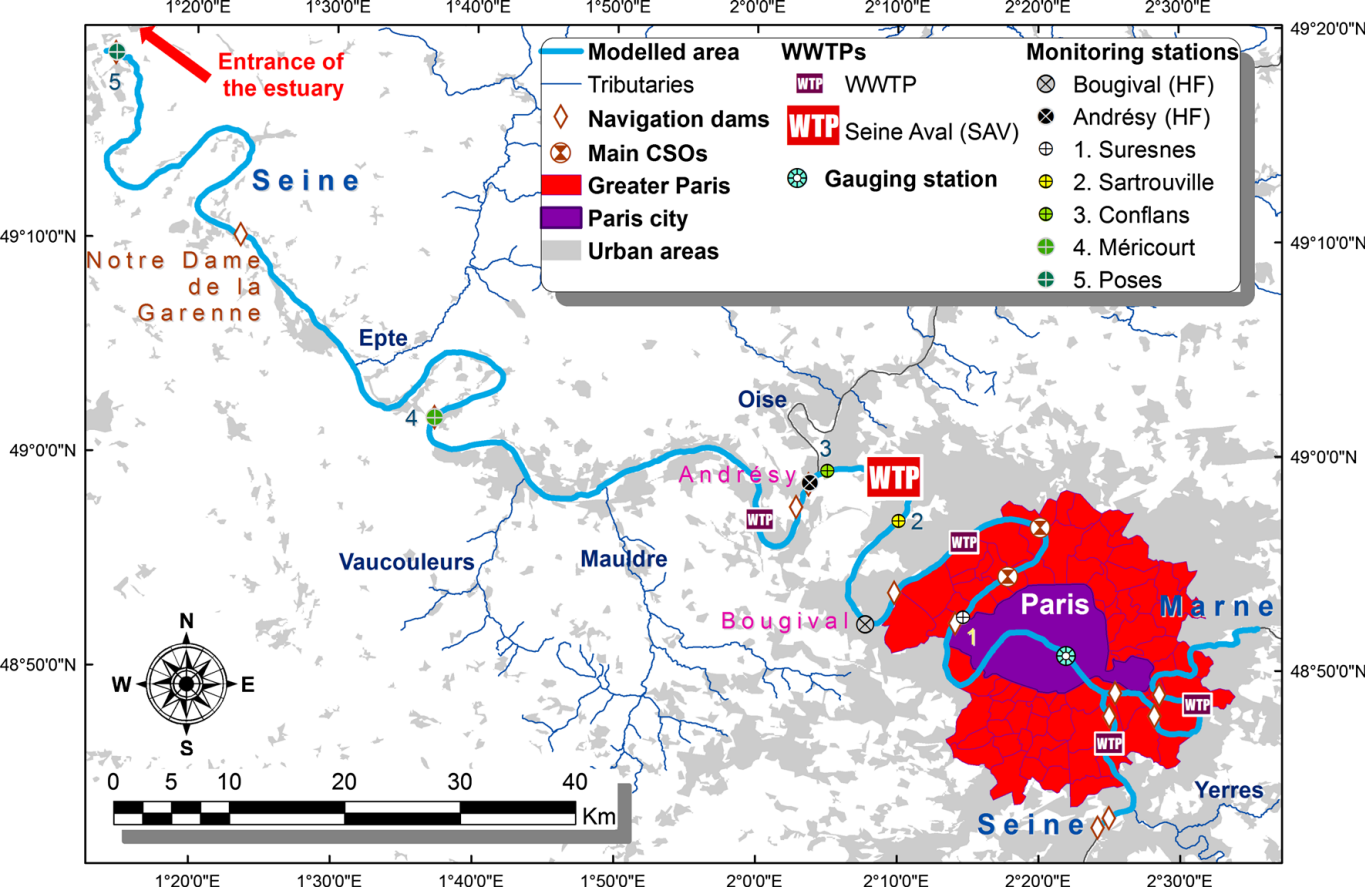
Study area, modeled river stretch, main tributaries and anthropogenic effluents, and locations of the CarboSeine high frequency monitoring stations

The Seine River’s hydro-biogeochemical functioning is simulated along a 220 km stretch (Fig. 1), from the Paris urban area to the entrance of the estuary (Poses), from January 2007 to December 2012. 17 km of the Marne River are also represented. The study area is located downstream from the large agricultural lands of the Seine and Marne river basins, which constitute important diffuse sources of nutrients. The Paris urban area exerts a high pressure on the receiving environment, notably through its large effluents. Indeed, this area bears almost one fifth of the total French population on less than 3 % of the territory (Billen et al. 2007). The waste water of the urban area’s population is collected in a combined sewer system and is treated in 5 WWTPs (see Fig. 1 for locations). Among these WWTPs, Seine Aval (SAV), which is located 70 km downstream from the Seine-Marne confluence in Paris, has the largest treatment capacity and treats the effluents of over 5 million equivalent inhabitants. It has a mean water discharge of 19 m ^3^⋅*s* ^−1^ for 2007–2012, which corresponds to about 15 % of the Seine River discharge in Paris during low flow periods. During large rain events, the combined sewer system may be saturated and can overflow through many stormwater discharge pipes (Even et al. 2004; 2007). These overflows constitute large inputs of sediments, organic matter and nutrients to the Seine River. The major combined sewer overflow (CSO) outlets are located 30-40 km downstream from Paris (Fig. 1). The Seine River’s mean daily discharge in Paris downstream from the Seine-Marne confluence is 310 m ^3^⋅*s* ^−1^. Along the studied stretch, the Seine River has two major tributaries: the Marne River and the Oise River. On average, each of these tributaries account for one fifth to one fourth of the daily discharge at Poses (440 m ^3^⋅*s* ^−1^). In addition to the Oise River, 3 smaller tributaries are accounted for as lateral boundary conditions.

Upstream water quality data of the Seine, Marne, and Oise rivers are provided at a daily time step by the public drinking water company of the Paris urban area (SEDIF), except for chl *a* concentrations. Weekly chl *a* measurements, provided by the public sewage company of the Paris urban area (SIAAP), are used as boundary conditions. 15-min time step chl *a* concentrations, which were acquired by the SEDIF, are also available at the upstream boundary of the simulated Marne River stretch for the 2011–2012 period. The quality of the three smaller tributaries is monitored at a lower frequency by the national river monitoring network (RCS). River daily discharges originate from the national Banque HYDRO database (www.hydro.eaufrance.fr). Daily measurements of the water flow and quality of the urban area’s five WWTP effluents are provided by the SIAAP. 151 CSOs and 15 small dry weather effluents are also taken into account as lateral boundary conditions.

### Validation of the model at short time scales

The model has been validated and applied to numerous case studies on the Seine River (Even et al. 1998; 2004; 2007; Vilmin et al. 2015b, a; Raimonet et al. 2015) or on smaller streams of the Seine River basin (Flipo et al. 2004; Flipo et al. 2007) at daily to pluri-annual time scales.

The recent implementation of high frequency monitoring stations (CarboSeine research program, see Fig. 1) allows for the validation of the model at shorter time steps. The CarboSeine network was set up to deepen our understanding of the biogeochemical functioning of the Seine river downstream from the Paris urban area at small time scales (Escoffier 2014). Among other parameters, it provides PO$_{4}^{3-}$ concentration measurements at a 4-h time step and chl *a* and O _2_ concentration measurements at a 15-min time step through optical sensing technologies (Escoffier et al. 2015; 2016).

**Fig. 2:**
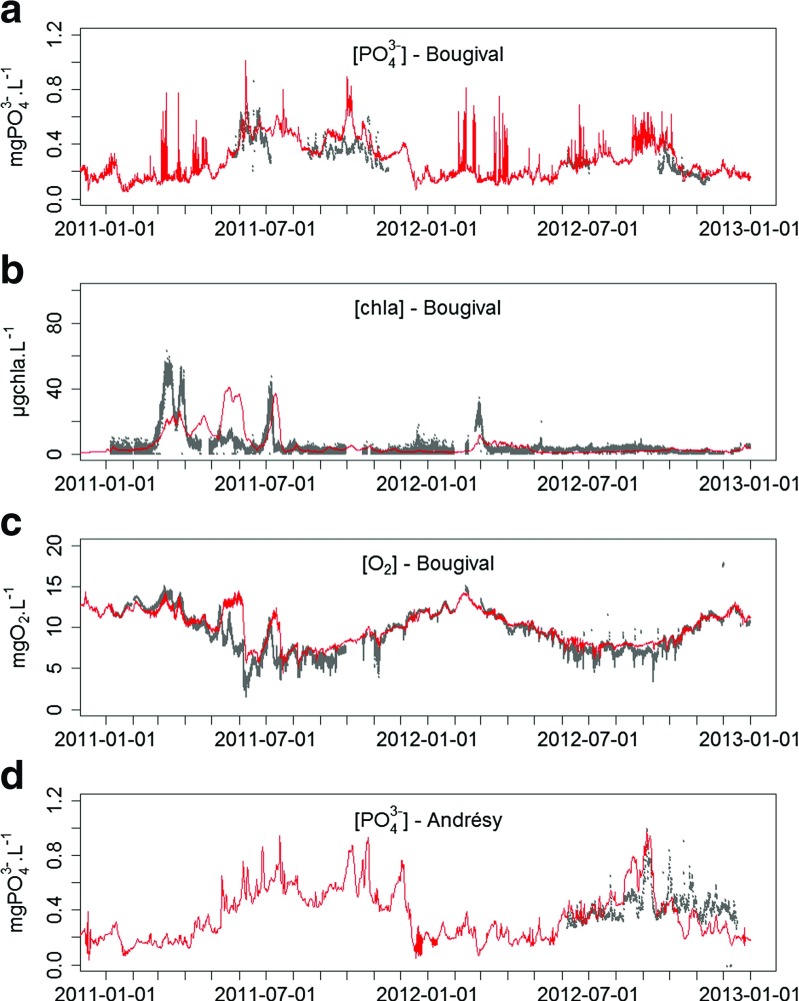
Measured (gray dots) and simulated concentrations (red line) of a) PO$_{4}^{3-}$ , b) chl *a* and c) O _2_ at Bougival and of d) PO$_{4}^{3-}$ at Andrésy (see Fig. 1 for locations of the monitoring stations)

The model results are compared graphically to the available time series recorded at the Bougival and Andrésy CarboSeine stations for the 2011–2012 period (Fig. 2). Statistical criteria—mean concentrations, standard deviations, correlation between simulated and measured time series, bias, and root mean square error (RMSE) 1—are also calculated to assess the model’s accuracy (Table 2). Mean and standard deviations of the simulated time series are calculated only for the values simulated at the measurement dates in order to be directly comparable with the characteristics of the measured time series. This validation is performed to ensure that ProSe can provide consistent high frequency time series to describe the pluri-annual water quality of the Seine River.

**Table 2:** Statistical comparison of simulated concentrations and measured time series at the CarboSeine monitoring stations

Criterion	Bougival			Andrésy
	PO$_{4}^{3-}$	chl *a*	O _2_	PO$_{4}^{3-}$
	mgPO$_{4}^{3-}\cdot \textit {L}^{-1}$	*μ*gchl *a* ⋅*L* ^−1^	mgO$_{2}\cdot \textit {L}^{-1}$	mgPO$_{4}^{3-}\cdot \textit {L}^{-1}$
*N* obs.	1982	60665	60902	1220
Mean sim.	0.37	5.78	9.83	0.42
Mean obs.	0.31	4.83	9.19	0.42
Std sim.	0.14	8.16	1.99	0.17
Std obs.	0.12	8.12	2.45	0.11
Correlation (adim.)	0.77	0.51	0.85	0.54
Bias	0.06	0.96	0.64	-0.01
RMSE	0.11	8.08	1.44	0.15

PO$_{4}^{3-}$ concentrations exhibit a high temporal variability. Concentrations can vary by a factor of 2 to 3 in less than 48 h. The ProSe model provides good estimates of PO$_{4}^{3-}$ concentrations at Bougival and Andrésy (Fig. 2a and d). The mean PO$_{4}^{3-}$ concentration is slightly overestimated at Bougival (mainly during the months of october and november 2011). The average differences between simulated and measured concentrations equal +21 % and -1 % at Bougival and Andrésy, respectively (Fig. 2a and d, Table 2). The simulated PO$_{4}^{3-}$ concentration time series are fairly well correlated to the measurements, with correlation coefficients of 0.77 (*p* value <10^−5^) at Bougival and 0.54 at Andrésy (*p* value=0.09).

Despite the loose upstream concentration data on the Seine River, the model outputs match the observed chl *a* dynamics at Bougival (Fig. 2b). Mean concentration is overestimated by the model (5.78 versus 4.83 *μ*gchl *a* ⋅*L* ^−1^), while the standard deviation of chl *a* concentrations is properly assessed (8.16 versus 8.12 *μ*gchl *a* ⋅*L* ^−1^). Measured and simulated chl *a* concentrations are rather well correlated, with a correlation coefficient of 0.51 (*p* value <10^−5^).

Seasonal and short term O _2_ dynamics are also properly simulated (Fig. 2c). The increases in concentration during algae blooms (March 2011 and July 2011) match the measured time series. The simulated O _2_ drops (usually linked to CSO events) are also well synchronized with the observed drops. Yet, the model tends to overestimate the minimum concentration values during these drops. The mean concentration and the standard deviation of the O _2_ concentrations are properly estimated by the model (9.83 versus 9.19 mgO$_{2}\cdot \textit {L}^{-1}$ and 1.99 versus 2.45 mgO$_{2}\cdot \textit {L}^{-1}$, respectively). Simulated and measured concentrations at Bougival in 2011–2012 are highly correlated, with a correlation coefficient of 0.85 (*p* value <10^−5^).

These results show that the model inputs and the formalisms used to represent the various biogeochemical processes allow for the estimation of the variations in water quality at short to seasonal time scales. Despite some identified discrepancies with the measured time series, we admit in the remaining of the paper that the ProSe model outputs can be used to mimic the high frequency functioning of the Seine River for a pluri-annual period of time.

### Seine River water quality from the Paris urban area to the estuary

#### Estimation of water quality indicators along the studied stretch

**Fig. 3:**
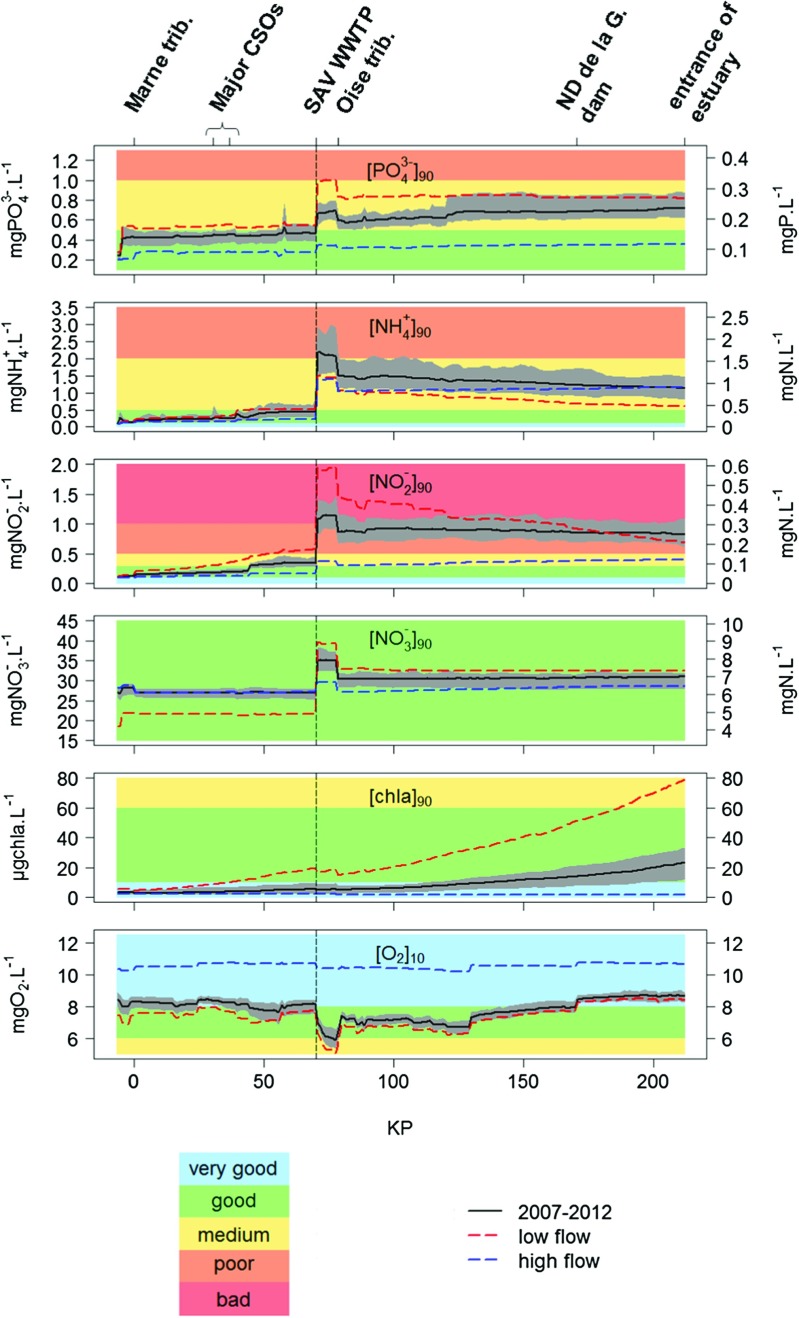
Longitudinal profiles of the WFD indicators for the studied variables (black solid line) and ranges of the calculated indicators for a sampling time step of 60 days (gray area). The red and blue dashed lines correspond to the indicators calculated for low flow and high flow periods only

In the remaining of the paper, the different locations are given as kilometer points (KP). This corresponds to curvilinear distances from the Seine-Marne confluence in Paris, in the direction of the Seine River’s flow. For each variable *i* (PO$_{4}^{3-}$, NH$_{4}^{+}$, NO$_{2}^{-}$, NO$_{3}^{-}$, chl *a* and O _2_), results are presented as graphs of longitudinal profiles of the corresponding water quality indicator *I* (pluri-annual 90 % quantiles of PO$_{4}^{3-}$, NH$_{4}^{+}$, NO$_{2}^{-}$, NO$_{3}^{-}$ and chl *a* concentrations—[PO$_{4}^{3-}$] _90_, [NH$_{4}^{+}$] _90_, [NO$_{2}^{-}$] _90_, [NO$_{3}^{-}$] _90_ and [chl *a*] _90_—and pluri-annual 10 % quantile of O _2_ concentrations—[O _2_] _10_) calculated for daily and for the recommended 60-day time step samplings (Fig. 3). For statistical relevancy, indicators are calculated over the whole 6-year time window.

The daily indicators are calculated on the simulated time series, which are re-sampled at a daily time step at noon (see Effect of the sampling hour for justifications). To estimate the ranges of the indicator values that can be obtained for a 60-day sampling, the indicators are estimated for all possible re-sampled time series (with a sampling at noon). Calculations are therefore performed for 60 different time series, which start on January 1, 2, 3, etc. For each indicator *I*, the span of the range of all 60 estimated indicator values (Δ*I*) can be assessed along the whole studied stretch.

Meybeck and Moatar (2012) highlighted that quality indicators can follow different trends, depending on the river flow. Also, depending on the dilution capacity of the flow, human effluents affect the downstream functioning in various ways. To assess the effect of the flow characteristics on the river’s water quality, the different water quality indicators are also calculated for low flow and high flow periods only (Fig. 3). As suggested by Vilmin et al. (2016), low flow periods correspond to the 30 consecutive driest days (based on the moving average of the daily discharge in Paris) of each calendar year, and high flow periods to the wettest consecutive 30 days of each hydrological year.

Numerical values are provided at 5 stations (Table 3): Suresnes (KP 24), Sartrouville (KP 65), Conflans (KP 78), Méricourt (KP 129) and Poses (KP 212). Suresnes is representative of the upstream part of the studied stretch. Sartrouville is located downstream from the major CSOs and upstream from the main WWTP, SAV. Conflans is located downstream from SAV and upstream from the Seine-Oise confluence. Finally, Méricourt is representative of the downstream sector and Poses is upstream from the last navigation dam at the entrance of the Seine River’s estuary, about 130 km downstream from the Seine-Oise confluence.

**Table 3:** Water quality status, optimal sampling time step, range of calculated indicator values for this optimal sampling time step and for a 60-day time step, and proportion of seasonal variability in total variability (season. var.) for all studied variables at five stations

			Suresnes	Sartrouville	Conflans	Méricourt	Poses
PO$_{4}^{3-}$	Status		Good	Good	Medium	Medium	Medium
	${\Delta } t_{opt,PO_{4}^{3-}}$	Days	9	12	10	7	11
	[PO$_{4}^{3-}$] _90_ (Δ*t*_*opt*_)	mgPO$_{4}^{3-}\cdot \textit {L}^{-1}$	0.43–0.44	0.46–0.48	0.67–0.71	0.67–0.69	0.70–0.73
	[PO$_{4}^{3-}$] _90_ (60 days)	mgPO$_{4}^{3-}\cdot \textit {L}^{-1}$	0.35–0.48	0.39–0.57	0.60–0.80	0.60–0.86	0.63–0.88
	Season. var.	%	34.8	39.3	16.8	26.4	30.5
NH$_{4}^{+}$	Status		Good	Good	Poor	Medium	Medium
	${\Delta } t_{opt,NH_{4}^{+}}$	Days	4	5	2	3	2
	[NH$_{4}^{+}$] _90_ (Δ*t*_*opt*_)	mgNH$_{4}^{+}\cdot \textit {L}^{-1}$	0.22–0.23	0.45–0.45	2.06–2.09	1.35–1.40	1.10–1.15
	[NH$_{4}^{+}$] _90_ (60 days)	mgNH$_{4}^{+}\cdot \textit {L}^{-1}$	0.16–0.37	0.29–0.64	1.59–2.90	1.04–1.73	0.83–1.48
	Season. var.	%	7.9	8.4	5.9	3.5	10.1
NO$_{2}^{-}$	Status		Good	Medium	Bad	Poor	Poor
	${\Delta } t_{opt,NO_{2}^{-}}$	Days	6	6	5	6	7
	[NO$_{2}^{-}$] _90_ (Δ*t*_*opt*_)	mgNO$_{2}^{-}\cdot \textit {L}^{-1}$	0.17–0.18	0.34–0.36	1.15–1.18	0.89–0.93	0.81–0.84
	[NO$_{2}^{-}$] _90_ (60 days)	mgNO$_{2}^{-}\cdot \textit {L}^{-1}$	0.15–0.22	0.29–0.44	0.94–1.45	0.74–1.13	0.63–1.08
	Season. var.	%	33.1	22.8	4.9	2.1	0.0
NO$_{3}^{-}$	Status		Good	Good	Good	Good	Good
	${\Delta } t_{opt,NO_{3}^{-}}$	Days	37	32	20	21	31
	[NO$_{3}^{-}$] _90_ (Δ*t*_*opt*_)	mgNO$_{3}^{-}\cdot \textit {L}^{-1}$	26.1–27.4	26.2–27.5	34.1–35.4	29.9–31.4	30.3–31.8
	[NO$_{3}^{-}$] _90_ (60 days)	mgNO$_{3}^{-}\cdot \textit {L}^{-1}$	25.7–27.9	25.5–28.1	32.6–37.2	28.3–31.9	28.0–31.9
	Season. var.	%	73.3	71.8	6.6	6.3	7.5
chl *a*	Status		Very good	Very good	Very good	Very good	Good
	Δ*t*_*opt*,*chla*_	Days	5	4	4	4	2
	[chl *a*] _90_ (Δ*t*_*opt*_)	*μ*gchl *a* ⋅*L* ^−1^	3.36–3.49	5.34–5.49	5.60–5.65	9.16–9.41	23.12–23.55
	[chl *a*] _90_ (60 days)	*μ*gchl *a* ⋅*L* ^−1^	2.57–6.70	3.08–9.68	3.15–9.58	5.81–12.96	12.13–33.16
	Season. var.	%	23.4	23.7	23.8	28.0	20.5
O _2_	Status		Very good	Very good	Medium	Good	Very good
	${\Delta } t_{opt,O_{2}}$	days	46	51	15	20	66
	[O _2_] _10_ (Δ*t*_*opt*_)	mgO$_{2}\cdot \textit {L}^{-1}$	7.96–8.36	7.94–8.34	5.76–6.00	6.56–6.89	8.45–8.86
	[O _2_] _10_ (60 days)	mgO$_{2}\cdot \textit {L}^{-1}$	7.77–8.43	7.53–8.42	5.40–6.55	6.36–7.12	8.30–8.88
	Season. var.	%	90.9	90.8	79.0	71.7	82.8

#### Orthophosphate

**Fig. 4:**
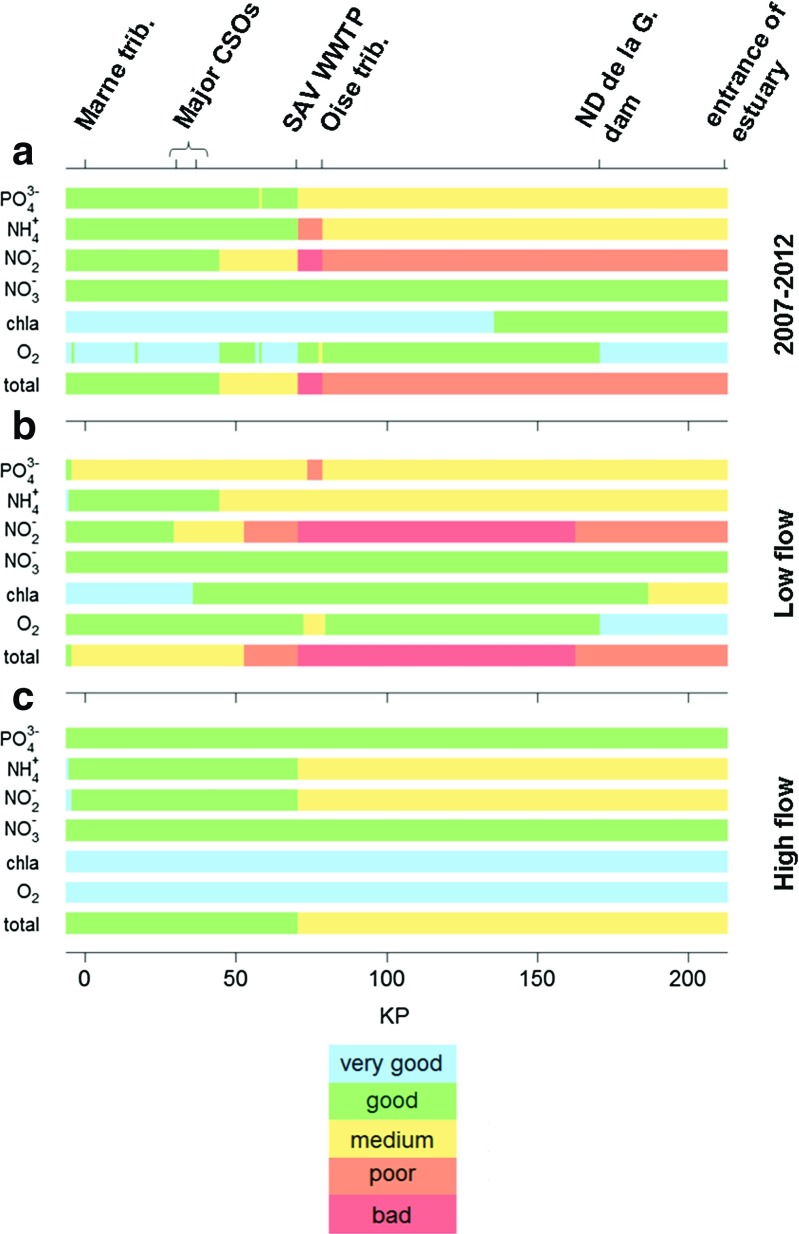
Longitudinal profiles of the water quality status of the Seine River estimated for a) the whole 2007-2012 period, b) low flows and c) high flows

The quality status of the Seine River in terms of PO$_{4}^{3-}$ is good from the Paris urban area to the SAV WWTP and shifts to medium downstream from the effluent (Figs. 3 and 4). In the study area, [PO$_{4}^{3-}$] _90_ is mainly affected by the WWTP effluents and the Oise River, downstream from which [PO$_{4}^{3-}$] _90_ decreases due to dilution. These discontinuities (WWTP effluents and Oise River) also induce changes in PO$_{4}^{3-}$ variability and in the uncertainties in the calculation of [PO$_{4}^{3-}$] _90_ . The proportion of seasonal variability in the total variability of PO$_{4}^{3-}$ concentrations decreases from 39 % upstream from SAV, at Sartrouville, to 17 % downstream, at Conflans (Table 3). Also, for a 60-day sampling, Δ[PO$_{4}^{3-}$] _90_ increases from 0.18 mgPO$_{4}^{3-}\cdot \textit {L}^{-1}$ just upstream from SAV to 0.21 mgPO$_{4}^{3-}\cdot \textit {L}^{-1}$ immediately downstream from the WWTP (Fig. 3).

During high flow periods, the quality in terms of PO$_{4}^{3-}$ is controlled by upstream agricultural inputs, and the [PO$_{4}^{3-}$] _90_ indicator is constant along the studied stretch (Fig. 3). On the contrary, during low flow periods, SAV has a significant effect on the water quality in terms of PO$_{4}^{3-}$ , which becomes poor downstream from the effluent and before dilution by the Oise River.

#### Ammonium

In terms of NH$_{4}^{+}$ , the quality status of the Seine River is good upstream from SAV (Figs. 3 and 4). The large NH$_{4}^{+}$ inputs from this effluent, which account for 75 % of all NH$_{4}^{+}$ inputs along the studied stretch, induce a deterioration of the downstream water quality that shifts to poor. After dilution by the Oise River, the [NH$_{4}^{+}$] _90_ values range within the boundaries of the medium quality status. SAV induces a small decrease in the proportion of seasonal variability in the total variability of NH$_{4}^{+}$ concentrations (from 8 to 6 %), which is already low upstream (Table 3). However, both the major CSOs, which contain large concentrations of NH$_{4}^{+}$, and the SAV WWTP lead to high increases in [NH$_{4}^{+}$] _90_ and in the uncertainty in its estimation (Fig. 3). For a 60-day sampling, Δ[NH$_{4}^{+}$] _90_ increases from 0.52 mgNH$_{4}^{+}\cdot \textit {L}^{-1}$ just upstream from SAV to 1.37 mgNH$_{4}^{+}\cdot \textit {L}^{-1}$ immediately downstream from the WWTP.

The quality of the Seine River in terms of NH$_{4}^{+}$ is clearly driven by anthropogenic effluents, whatever the hydrological conditions (Figs. 3 and 4). At the estuary, low flow [NH$_{4}^{+}$] _90_ values are however lower than high flow or pluri-annual values due to higher nitrification rates.

#### Nitrite

In terms of NO$_{2}^{-}$, the quality status of the Seine River is good upstream from the Paris urban area’s major CSOs (Figs. 3 and 4). Downstream from the CSOs, where NO$_{2}^{-}$ is produced by nitrification, the status shifts to medium. The SAV effluent constitutes 64 % of all NO$_{2}^{-}$ inputs along the studied stretch and induces a shift of the quality status in terms of NO$_{2}^{-}$ from medium to bad. 8 km downstream, NO$_{2}^{-}$ concentrations are diluted by the Oise River and the status becomes poor until the entrance of the estuary. As for PO$_{4}^{3-}$ and NH$_{4}^{+}$ , the different spatial heterogeneities induce significant changes in both [NO$_{2}^{-}$] _90_ values and uncertainties in their estimation (Fig. 3). SAV induces a clear decrease of the proportion of seasonal variability in the total variability of NO$_{2}^{-}$ concentrations (from 23 % at Sartrouville to 5 % at Conflans, see Table 3). For a 60-day sampling, Δ[NO$_{2}^{-}$] _90_ increases from 0.16 mgNO$_{2}^{-}\cdot \textit {L}^{-1}$ just upstream from SAV to 0.56 mgNO$_{2}^{-}\cdot \textit {L}^{-1}$ immediately downstream from the WWTP.

The quality of the Seine River in terms of NO$_{2}^{-}$ is controlled by anthropogenic effluents, but is also highly affected by flow conditions (Figs. 3 and 4). CSOs and the SAV effluent induce larger increases in [NO$_{2}^{-}$] _90_ during low flow periods due to higher NO$_{2}^{-}$ production in the river system. Indeed, low flow conditions coincide with periods of higher temperatures, which promotes nitrification activity (Raimonet et al. 2015).

#### Nitrate

From the Paris urban area to the entrance of the estuary, the Seine River’s quality status in terms of NO$_{3}^{-}$ is constantly good (Figs. 3 and 4). NO$_{3}^{-}$ mainly originates from the runoff from arable lands from the upstream agricultural drainage basins, which explains the large proportion of annual variability in the upstream sector (>70 % in Suresnes and Sartrouville, see Table 3). NO$_{3}^{-}$ concentrations are therefore less affected by urban effluents than those of the other nitrogen compounds (NH$_{4}^{+}$ and NO$_{2}^{-}$), even though the SAV effluent induces a clear increase in [NO$_{3}^{-}$] _90_ and a decrease in the proportion of seasonal variability of NO$_{3}^{-}$ concentrations (Fig. 3 and Table 3).

However, during low flow periods, when the runoff from agricultural lands is lower, SAV has a more visible effect on [NO$_{3}^{-}$] _90_, which is multiplied by 2 downsteam the effluent outlet, but remains within the range of the good quality status (Figs. 3 and 4).

#### Chlorophyll *a*

The water quality status in terms of chl *a* shifts progressively from a very good status to a good status along the studied stretch (Figs. 3 and 4). Contrary to the other studied variables, physical and anthropogenic heterogeneities do not exert any direct impact on [chl *a*] _90_. [chl *a*] _90_ and Δ[chl *a*] _90_ both increase along the river stretch. This can be explained by the fact that the intensity of the blooms increases, notably due to longer residence times. Even though there is no direct effect of anthropogenic effluents on [chl *a*] _90_, algal growth is promoted by the large inflows of nutrients, which contribute to the increase of the blooms’ intensity. The proportion of seasonal variability in the total variability of chl *a* concentrations does not seem to be affected by the different anthropogenic heterogeneities and remains in the range of 20-30 % along the studied stretch (Table 3).

Hydrology has a clear effect on the water quality status in terms of chl *a* , since algae blooms usually coincide with periods of low discharge (Garnier et al. 1995; Garnier and Billen 2007; Descy et al. 2012). During low flow periods, [chl *a*] _90_ can reach values corresponding to the medium quality status in the downstream sector of the study area, where high nutrient concentrations and long water residence times promote high algal growth (Figs. 3 and 4).

#### Dissolved oxygen

In terms of O _2_ and based on the 10 % quantiles calculated for the whole 2007–2012 period, the quality status of the Seine River fluctuates between the very good and good statuses upstream from SAV (Figs. 3 and 4). Downstream from SAV, the 10 % quantile drops rapidly and reaches the threshold of the medium status. Downstream from the Seine-Oise confluence, and after re-oxygenation at the Andrésy dam just downstream from the confluence (KP 79), [O _2_] _10_ values return to the good status. In the downstream section, the quantile values increase, and exceed the very good status threshold downstream from the Notre Dame de la Garenne dam (KP 170, see Fig. 1 for location). O _2_ concentrations exert a high seasonal variability that accounts for more than 70 % of their total variability along the studied stretch (Table 3).

At high flow, which coincide with colder periods, the water quality in terms of O _2_ is very little affected by anthropogenic heterogeneities (Figs. 3 and 4). Indeed, the O _2_ saturation concentration is higher and the high water velocities induce high re-aeration rates at the surface. At low flow, the O _2_ saturation concentration is lower, the flow has a lower dilution capacity and biological activity (i.e., respiration rates) is higher. During low flow periods, the quality status in terms of O _2_ therefore shifts to medium in the reach between SAV and the Seine-Oise confluence (Figs. 3 and 4).

#### Overall water quality for the 6 studied variables

Among the six studied variables, the overall water quality of the Seine River from the Paris urban area to the estuary is assessed in each model cell, based on the results for the most downgrading variable (Fig. 4). This analysis reveals that, among the studied variables, nitrogen compounds degrade the water quality along this river stretch. The overall quality is mostly driven by NO$_{2}^{-}$ concentrations. NO$_{2}^{-}$ is indeed the most degrading variable on average for 2007–2012 (Fig. 4a), especially at low flow downstream from the major CSOs (Fig. 4b). During high flow periods, the quality status of the Seine River is controlled by NH$_{4}^{+}$ and NO$_{2}^{-}$ concentrations (Fig. 4c).

For the 2007–2012 period, the quality shifts from good to medium downstream from the major CSOs. The most sensitive stretch is the reach between SAV and the Seine-Oise confluence, where the water quality status is bad. After dilution by the Oise River, the quality status becomes poor (Fig. 4a).

If we focus on low flow periods only, anthropogenic effluents have an even larger effect on the water quality of the river. The water quality is already medium downstream from the most upstream WWTP of the Paris urban area (KP -5, see Fig. 1). It is then driven by NO$_{2}^{-}$ concentrations downstream from the major CSOs until the entrance of the estuary (Fig. 4b).

Anthropogenic pressures have less effect on the water quality during high flow periods, when the Seine River and its tributaries have a greater dilution capacity, and when biological activity (notably nitrification) is lower. However, the water quality still shifts from a good status in the upstream area to a medium status downstream from SAV (Fig. 4c). Even for these flow conditions, the good status as defined by the WFD is not achieved downstream from the major effluents of the Paris urban area.

These results show that the water quality status, as defined by the WFD, is very sensitive to the fixed threshold values separating the different quality statuses, especially for the most downgrading variable (NO$_{2}^{-}$ in the present case). In the objective of a 75 % drop of the nitrogen inputs to the coasts, a decrease of the limit of the good quality status for NO$_{3}^{-}$ from 50 to 18 mgNO$_{3}^{-}\cdot \textit {L}^{-1}$ is currently being considered in France. With this new limit, the overall water quality status of the Seine River would already be bad upstream from the Paris urban area, due to the large upstream NO$_{3}^{-}$ inputs from agricultural lands.

Most water quality variables are also sensitive to the flow conditions. It would therefore be relevant to estimate the water quality indicators at a seasonal scale rather than at annual or pluri-annual scales, and define seasonal quality standards. Thereby, the effect of large anthropogenic effluents on important quality variables, such as O _2_, would be better assessed.

## Does an optimal sampling frequency exist?

### Effect of the sampling hour

Even though for variables with high daily variability the sampling hour can also affect the calculated statistical criteria (Scholefield et al. 2005; Wade et al. 2012; Halliday et al. 2012), its effect cannot be assessed through our modeling approach. Indeed, the models’ boundary conditions are informed at a daily time step, and the variability linked to sub-daily variations of inputs may therefore be underestimated. In an attempt to further assess the effect of this sub-daily variability, O _2_ high frequency (15 min) time series recorded at Bougival in 2011 are re-sampled at a daily frequency according to 12 different time stamps (every 2 h from 00:00 to 22:00). Boxplots of the re-sampled time series are calculated and compared in order to determine the effect of the sampling time.

**Fig. 5:**
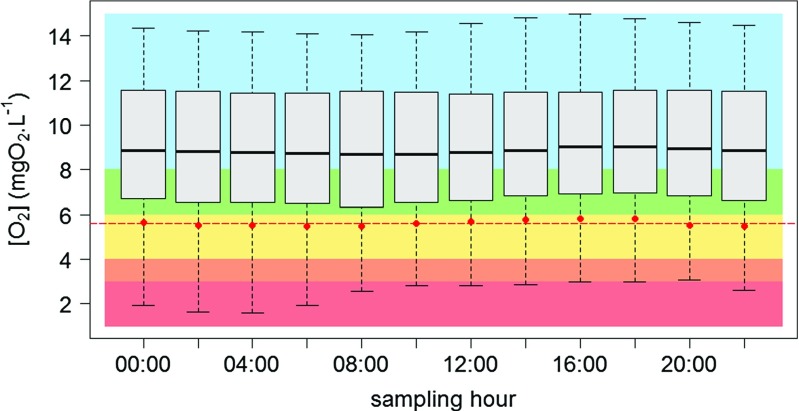
Effect of the sampling timestamps on O _2_ variability at Bougival in 2011. Horizontal markers inside the boxes indicate median concentration; boxes represent the 25 % and 75 % quantiles; horizontal markers outside the boxes indicate the entire ranges of daily O _2_ concentrations. Red dots represent the corresponding 10 % quantiles; the red dashed line indicates the mean 10 % quantile for all timestamps

The daily re-sampling of the high frequency time series of O _2_ concentrations at Bougival in 2011 provides an overview of the daily trends in O _2_ concentrations in an urbanized environment (Fig. 5). In 2011 at Bougival, [O _2_] _10_ is close to the threshold between the good and medium water quality levels (6 mgO$_{2}\cdot \textit {L}^{-1}$). The comparison of the [O _2_] _10_ values for the different sampling hours reflects the effect of sub-daily biological dynamics (i.e., O _2_ primary production during light periods), since the values obtained for afternoon samplings are systematically higher than those obtained in the morning. The maximum daily amplitudes of [O _2_] _10_ can reach 0.5 mgO$_{2}\cdot \textit {L}^{-1}$, which corresponds to 25 % of the span of the interval defining the good quality status (6-8 mgO$_{2}\cdot \textit {L}^{-1}$). The effect of the sampling hour can thus be significant for the assessment of the water quality status, especially since the [O _2_] _10_ values fluctuate around the good/medium threshold. The effect of the sampling time step would be even greater for more eutrophic rivers, as the Loire River, that can exert daily amplitudes of O _2_ concentrations of several mgO$_{2}\cdot \textit {L}^{-1}$ (Minaudo et al. 2015).

The analysis of the daily O _2_ fluctuations indicates that an average situation is reached around noon every day. For the remaining of the paper, we therefore consider that the water quality at noon is representative of the daily quality. The current analysis of the effect of sub-daily fluctuations indicates that our further estimate of O _2_ water quality status will entail a small uncertainty of ±0.25 mgO$_{2}\cdot \textit {L}^{-1}$.

### Assessment of optimal sampling frequencies

We assess the optimal sampling frequencies for the monitoring of PO$_{4}^{3-}$, NH$_{4}^{+}$, NO$_{2}^{-}$, NO$_{3}^{-}$, chl *a* and O _2_ for an accurate estimation of the 6 associated WFD water quality indicators.

**Fig. 6:**
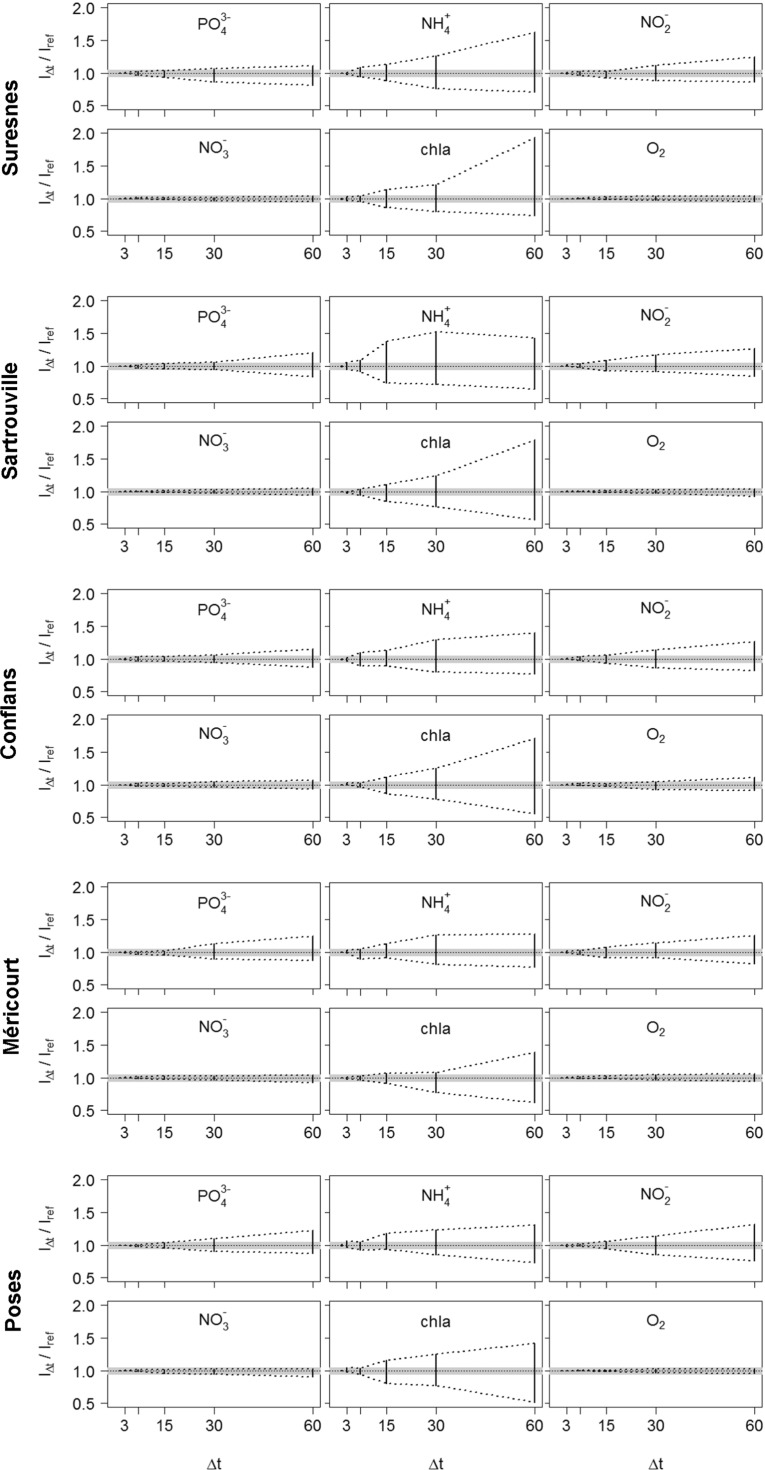
Ranges of the uncertainties in the estimated quality indicators for different sampling time steps. The gray area corresponds to the 5 % confidence interval around the daily reference value

To quantify the effect of the sampling time step on the estimation of water quality indicators, the total simulated 2007-2012 time series are numerically re-sampled at every time step ranging from 1 to 90 days (at noon). The indicators calculated on the daily time series are considered as the reference (*ref*). For each time step and for each indicator *I*_Δ*t*_, the range of possible estimated values is assessed as described in “Estimation of water quality indicators along the studied stretch” for a 60-day time step. The ranges of the calculated values of *I*_Δ*t*_/ *I*_*ref*_ are plotted at Suresnes, Sartrouville, Conflans, Méricourt and Poses for different sampling time steps (3, 7, 15, 30, and 60 days, see Fig. 6). Not surprisingly, the uncertainty grows with the increase of the sampling time step. Figure 6 indicates that the relative increase is linked to (i) the variable considered and (ii) the sampling location. It also clearly indicates that the optimal sampling frequency is not unique but rather depends on more subtle criteria.

**Fig. 7:**
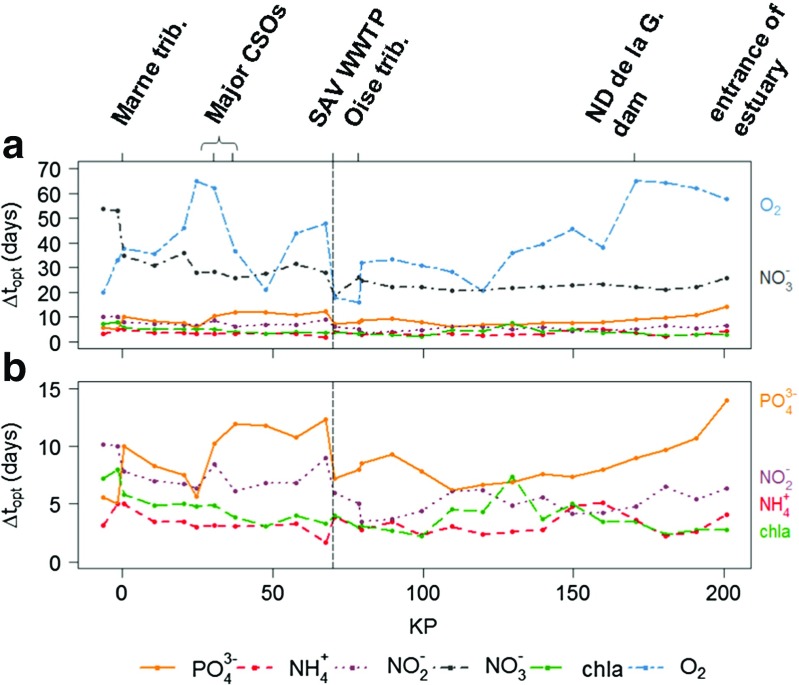
Longitudinal profiles of the optimal sampling time steps for the studied variables: a) for all studied variables, b) zoom on the most variable compounds

In the present study, we define as an accurate estimate of the different WFD indicators, an estimate with less than 5 % error. We therefore assess the minimum frequency (maximum time step) needed to obtain less than % error on the indicator estimation (i.e., Δ*I*_Δ*t*_ is inferior to 5 % of *I*_*ref*_). For the variable *i*, this time step is noted Δ*t*_*opt*,*i*_. Longitudinal profiles of the estimated Δ*t*_*opt*,*i*_ are plotted in Fig. 7.

It is important to note that the efficiency of the present method depends on the ability of the model to reproduce the dynamics of the studied variables. In fact, discrepancies between the simulated temporal variability of a variable and its variability in the environment can lead to uncertainty in the estimated optimal sampling frequency. For instance, the simulated O _2_ time series at Bougival (standard deviation of 1.99 mgO$_{2}\cdot \textit {L}^{-1}$) is less variable than the observed one (standard deviation of 2.45 mgO$_{2}\cdot \textit {L}^{-1}$, see Table 2). The optimal sampling time step defined on the simulated time series might therefore be overestimated. In the same way, the model slightly overestimates the variability of PO$_{4}^{3-}$ concentrations at Bougival and Andrésy (Table 2), which can lead to a little underestimation of the optimal sampling time step.

Also, the defined “optimality” may depend on the period of the study. Kusmulyono and Goulter (1995) showed that, depending on the length of the available record, the time window for which the water quality indicators are estimated can have a significant impact on the accuracy of the prediction. Here, in order to limit this impact, we chose to assess the water quality of the Seine over a 6-year period of time, which comprises years that are contrasted in terms of hydrology (Vilmin et al. 2015b).

### Optimal sampling frequency depends on the monitored variable and its drivers

Lázslo et al. (2007) showed that the optimal sampling frequency depends on the sampling location (hydro-morphological characteristics, presence of anthropogenic effluents, *etc.*), and on the measured variable and its variability in the receiving environment. Our results confirm these assertions. It is not possible to define one single optimal sampling frequency for water quality monitoring as defined by the WFD. However, it is possible to define for each variable and at different locations an optimal sampling time step, which allows to capture its variability in the environment (Fig. 7). At this optimal sampling time step, the acquired concentration time series is representative of the system’s functioning and accurate statistical indicators can then be calculated. The optimal time steps depend on the temporal variability of each parameter, which is concomitantly affected by natural processes and local anthropogenic influences.

For variables, which mainly originate from urban sources (PO$_{4}^{3-}$, NH$_{4}^{+}$, NO$_{2}^{-}$), optimal sampling time steps depend on the location of the sampling site with respect to the major anthropogenic effluents (i.e., CSOs, WWTPs). In fact, the major effluents lead to a sharp increase of their variability in the receiving environment and higher sampling frequencies are thus needed downstream. Raimonet et al. (2015) already highlighted the effect of the sampling frequency on river environmental assessment. They showed that, downstream from the major effluent of the Paris urban area, monthly sampling does not permit to account for the high variabilities of NH$_{4}^{+}$ and NO$_{2}^{-}$. The present study confirms that a 60-day or even monthly sampling time step is not sufficient to estimate the water quality indicators for the compounds originating from urban effluents (Fig. 6). A weekly sampling allows for the assessment of [PO$_{4}^{3-}$] _90_ along the whole studied stretch. The median optimal sampling frequency for an accurate estimation of [PO$_{4}^{3-}$] _90_ is indeed 10 days upstream from SAV and 8 days downstream from the WWTP (Fig. 7). Smaller sampling time steps are needed for NH$_{4}^{+}$ along the whole studied stretch and for NO$_{2}^{-}$ downstream from SAV to completely account for these variables’ variabilities. For an estimation of [NH$_{4}^{+}$] _90_ and [NO$_{2}^{-}$] _90_ with less than 5 % error, sampling time steps of 1-8 and 2-14 days are needed, depending on the location (Fig. 7). The median optimal time step for NH$_{4}^{+}$ monitoring is 3 days both up- and downstream from SAV. It is 7 days upstream from SAV and 5 days downstream from SAV for NO$_{2}^{-}$.

For the variables that display a larger seasonal variability, the optimal sampling frequency is much lower. For these variables, a monthly to 60-day time step is enough to accurately assess WFD indicators in most locations that are far enough from pollution sources. In the Seine River, this is the case for NO$_{3}^{-}$ and, to a lesser extent, for O _2_ concentrations. NO$_{3}^{-}$ mainly originates from the runoff over upstream agricultural lands. NO$_{3}^{-}$ concentrations are therefore highly correlated to the hydrology, which explains the high proportion of annual variability in the total variability of NO$_{3}^{-}$ concentrations (>70 % upstream from SAV, see Table 3). Along the studied stretch, optimal sampling time steps for an accurate estimation of [NO$_{3}^{-}$] _90_ range from 11 to 59 days (Fig. 7). A 25-day sampling time step, which corresponds to the median value, can be considered sufficient for a good estimation of the [NO$_{3}^{-}$] _90_ indicator. O _2_ variations are also mainly seasonal, since they are strongly controlled by saturation that is a function of temperature. Indeed, seasonal variability accounts for more than 70 % of the total variability of O _2_ concentrations at the five stations (Table 3). Along the studied stretch, [O _2_] _10_ can be estimated with less than 5 % error with a 14–83-day sampling time step (Fig. 7). Since SAV induces a higher variability of O _2_ concentrations, more frequent measurements are necessary between the effluent and the Seine-Oise confluence, where a median time step of 15 days is needed.

Finally, the response of biotic variables, such as chl *a*, to anthropogenic pollution is not direct. Therefore, the [chl *a*] _90_ indicator does not directly reflect the impact of human pressure, and major effluents do not directly affect the optimal sampling frequency for chl *a* monitoring. Yet, these heterogeneities certainly contribute to the slow longitudinal variations in [chl *a*] _90_ by inducing changes in the environment’s characteristics (i.e., water flow, nutrient concentrations). Along the studied stretch, an accurate estimation of [chl *a*] _90_ requires sampling time steps inferior to 1 week (Fig. 7). The assessed optimal sampling time steps range from 1 to 9 days, with a median value of 4 days. Due to the highly transient character of algae blooms, which occur maximum 2 to 3 times a year, the estimation of [chl *a*] _90_ is very sensitive to the sampling frequency (Fig. 6) and chl *a* concentrations need to be monitored at small time steps to be sure to capture the peaks.

### Optimal sampling frequency depends on the monitoring goals

The optimal sampling frequency also depends on which information is expected from the data. Formulating the specific objectives of the monitoring strategy is probably the most important and most difficult step in the entire monitoring process (Lettenmaier 1979; Timmerman et al. 2000). Monitoring networks can have a lot of various objectives and they usually combine several of them. The present work deals with water quality surveillance monitoring, which aims at assessing long-term changes and providing baseline data on river basins (Allan et al. 2006). Yet, when the good status is not achieved, additional monitoring is necessary to assess the causes of such failure and the effect of remediation strategies (Allan et al. 2006).

The second element that constrains the design of monitoring strategies is data analysis (Timmerman et al. 2000). It is important to determine how the information extracted from the measurement data should be presented and the level of precision to be included in this information (Timmerman et al. 2000). Therefore, statistical design criteria must be established and the variables under study need to be characterized (variation in quality, seasonal impacts, *etc.*) (Ward et al. 1986). In the present study, we focus on the estimation of WFD water quality indicators. Yet, what is considered as an accurate estimation of these indicators also needs to be defined. We give here the example of an acceptable 5 % error.

However, a less than 5 % error does not always guarantee a good assessment of the quality status, when the variable’s values are close to the threshold between two quality classes. For example, upstream from SAV and immediately downstream from the Seine-Oise confluence, [PO$_{4}^{3-}$] _90_ values are close to the good/medium statuses threshold (Fig. 3). A slight error in the estimation of [PO$_{4}^{3-}$] _90_ can thus lead to an error in the assessment of the water quality status. In the same way, for an accurate estimation of the water status regarding NH$_{4}^{+}$ and NO$_{2}^{-}$, close attention should be paid to the reach downstream from the major CSOs and upstream from SAV, where quantile values are close to the good/medium statuses threshold (Fig. 3). Regarding O _2_, 6 km downstream from the SAV WWTP, a 14-day sampling is enough to assess [O _2_] _10_ with less than 5 % error. Yet, a 3-day sampling time step is necessary for a reliable estimation of the quality status, due to the large drop of oxygen concentrations that causes the [O _2_] _10_ values to reach the good/medium statuses thresholds (Fig. 3).

This analysis points out that an adaptative sampling time step is needed for a proper survey of water quality. The idea of a single optimal sampling time step for water quality monitoring is obsolete, especially with the recent improvements in sensor technologies, which allow for the definition of various sampling frequencies for different variables at a single location without unrealistically increasing the human constraint. For instance, the CarboSeine stations record chl *a* at a 15-min time step and PO$_{4}^{3-}$ at a 4-h time step. The use of these new technologies increases flexibility and opens the door to the development of more powerful monitoring networks. As discussed below, the design and the operation of such monitoring networks can benefit from the simultaneous development of high-resolution numerical models.

## Use of modeling tools to optimize and support water quality assessment

The present study shows how a modeling tool can be used to support the design of monitoring strategies. Beforehand, the model must be validated as an acceptable representation of the system (based on the comparison with historical data) (Radford and West 1986). As presented here, models can be used to help in the definition of optimal sampling time steps. Models can also provide information on the spatial discontinuities and their distance of impact. Moreover, they allow for the identification of river reaches, which are the most sensitive to human pressure (from SAV to the Seine-Oise confluence, in the present case). They can, therefore, also be used to decide on the best sampling locations.

The use of adequate sampling frequencies at relevant sampling sites would minimize the uncertainties in the estimated water quality indicators. With too low frequencies, concentration peaks may not be well captured and the calculation of the indicators may be flawed. We showed that the estimation of water quality indicators on data from 60-day sampling can exert high uncertainties. However, monitoring all water bodies at optimal resolution for an accurate estimation of WFD indicators is unrealistic, because of logistical and financial limitations. A validated model can provide more accurate statistical criteria than too loose data. For example, Radford and West (1986) showed that an estuarine predictive model produced better estimates of mean pollutant concentrations than those obtained from observations alone. Therefore, the fine spatio-temporal resolution that can be achieved by simulation models should be used to complete the information contained in measured data. High frequency calculated time series of highly variable compounds could be used to complete data measured at a lower frequency. This can be achieved with interpolation methods such as co-kriging.

Our method also allows for the assessment of the uncertainties in the statistical criteria derived from the data, which are related to the sampling frequency. Indeed, it provides for every time step a range of possibly assessed indicator values. The multiple re-sampling of high frequency simulated time series we used could also be employed for a stochastic analysis of the water quality, and density probability functions of the water quality status could be derived at given locations. In this way, instead of providing a unique quality status that can be erroneous, the likelihoods of the water body’s different status classifications could be assessed (Hering et al. 2010).

Finally, modeling tools can provide, in addition to high-resolution concentrations, information on the interactions between the different quality variables, on the river biogeochemical transformations, on the system’s metabolism *etc.* This information is essential to understand the drivers of the health of water bodies. WFD indicators alone do not permit such an understanding of the functioning of river systems. For fast varying variables that originate from urban point sources, WFD indicators exhibit the impact of effluents on downstream quality if the sampling is performed at an adequate frequency. For variables with high seasonal variability, quantiles do not capture the effect of peaks induced by human disturbances, since the seasonal variability may be larger than these peaks. Along the studied stretch of Seine River, this is the case for O _2_, which is certainly one of the most integrative water quality variables. O _2_ concentrations are indeed affected by (and affect) many biogeochemical processes (Nimick et al. 2011). For example, the effect of major CSOs, which can induce large drops of O _2_ concentrations during highly transient events, is not visible on the pluri-annual [O _2_] _10_ longitudinal profile (Fig. 3). For these variables, water quality indicators could be calculated not on the variables themselves, but on transformed variables that do not exert the natural variability anymore. In the case of O _2_, several authors showed that river metabolism could be used as a functional metric for the assessment of river health (Fellows et al. 2006; Young et al. 2008; Trimmer et al. 2012). Yet, the monitoring of river metabolism requires high frequency data (Escoffier et al. 2016) and may be too expensive to maintain over large spatial scales. A validated modeling tool such as ProSe can provide good estimates of these metrics and can be used to complete the information obtained by monitoring networks.

All our results emphasize the fact that, around major urban areas, the joint development of water quality modeling tools and high frequency monitoring networks should be favored by water authorities in order to build up preservation strategies for water resources.

## Summary and conclusion

In the present paper, we define, through a modeling approach, optimal sampling time steps to accurately assess different water quality indicators defined by the WFD. We focus on the case of a highly human-impacted 220 km stretch of the Seine River.

Our results show that, in large rivers that are subject to high urban pressure, a 2-month time step is not sufficient for the monitoring of variables that mainly originate from urban effluents (PO$_{4}^{3-}$, NH$_{4}^{+}$ and NO$_{2}^{-}$). For the latter variables, a sampling time step of 1 week or less, notably for NH$_{4}^{+}$, is needed. The monitoring of chl *a* also requires a sampling time step inferior to one week, due to the highly transient character of bloom events. WFD indicators for NO$_{3}^{-}$ and O _2_, which have significant seasonal variations, can be assessed in an acceptable way with monthly data. Yet more measurements may be needed for O _2_ concentrations downstream from major effluents. However, our results show that these indicators are sensitive to the flow conditions. It might therefore be relevant to assess the quality of water bodies at a seasonal scale or, at least, in conjunction with hydrological conditions.

The method we apply here can be used for the design of monitoring strategies. Beforehand, in order to efficiently meet the monitoring project’s expectations, it is essential to identify (i) which questions need to be addressed and (ii) how the answer should be presented (i.e., criteria and precision, Timmerman et al. (2000)). A modeling tool, which provides reliable estimates of the variabilities of the studied variables, can then be used to assess the effect of the sampling frequency on the estimation of the water quality indicators and to select the optimal time step for each variable. This optimal time step depends on the location of the sampling and needs to be revised in case of changes in some of the drivers of the water quality variables.

We focus here on six water quality variables of the ecological status, whose values and variations are correctly assessed by the used model. Our method could also be applied to other pollutants, as long as a model that accurately reproduces their dynamics in the environment is available. For example, developments in the modeling of nonylphenols or persistent organic pollutants in river systems (Cladière et al. 2014) will be helpful to support the design of monitoring strategies for these contaminants.

This work highlights the important role that modeling tools can play in monitoring network design and in providing additional information for a better quality assessment of water bodies. It also suggests that the water quality survey in urban areas could be significantly improved by the coupled development of automated, adjustable time step monitoring networks and numerical models, such as ProSe .

## Footnotes

1 bias=${\frac {1}{N}{\sum \nolimits _{i}^{N}} }(S_{i}-O_{i})$ and RMSE=$\sqrt {\frac {1}{N}{\sum \nolimits _{i}^{N}} (S_{i}-O_{i})^{2}}$, where *S* and *O* are the simulated and observed time series and *N* is the number of observations.
